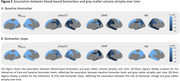# Longitudinal associations between blood‐based biomarkers of Alzheimer's disease and MRI‐measured cortical thickness and grey matter volume in subjective cognitive decline

**DOI:** 10.1002/alz70856_100846

**Published:** 2025-12-25

**Authors:** Calvin Trieu, Ellen Dicks, Mardou S. S. A. van Leeuwenstijn, Lisa‐Marie Schlüter, Lynn Boonkamp, Frederik Barkhof, Wiesje M. van der Flier, Charlotte E. Teunissen, Argonde C. van Harten

**Affiliations:** ^1^ Alzheimer Center Amsterdam, Vrije Universiteit Amsterdam, Amsterdam UMC location VUmc, Amsterdam, Noord‐Holland, Netherlands; ^2^ Amsterdam Neuroscience, Neurodegeneration, Amsterdam, Noord‐Holland, Netherlands; ^3^ Neurochemistry Laboratory, Amsterdam Neuroscience, Program Neurodegeneration, Amsterdam UMC, Vrije Universiteit Amsterdam, Amsterdam, Noord‐Holland, Netherlands; ^4^ Neurochemistry Laboratory, Department of Laboratory Medicine, Amsterdam UMC, Vrije Universiteit Amsterdam, Amsterdam Neuroscience, Amsterdam, Netherlands; ^5^ Queen Square Institute of Neurology and Centre for Medical Image Computing, University College London, London, Greater London, United Kingdom; ^6^ Department of Radiology and Nuclear Medicine, Vrije Universiteit Amsterdam, Amsterdam University Medical Center, location VUmc, Amsterdam, Netherlands; ^7^ Amsterdam Neuroscience, Neurodegeneration, Amsterdam, Netherlands; ^8^ Alzheimer Center Amsterdam, Neurology, Vrije Universiteit Amsterdam, Amsterdam UMC location VUmc, Amsterdam, Netherlands

## Abstract

**Background:**

Blood‐based biomarkers have been established as reliable markers of Alzheimer's disease (AD)‐related pathology in individuals with subjective cognitive decline (SCD). It remains unclear how these biomarkers and their longitudinal changes are associated with gray matter atrophy in SCD. This longitudinal study investigates the relationship between (changes in) blood‐based biomarkers and changes in cortical thickness and hippocampal volume in individuals with SCD.

**Method:**

We included 167 individuals with SCD (49 amyloid‐positive [A+] and 118 amyloid‐negative [A−]) who underwent biennial blood sampling (*n* = 484) and repeated imaging over a follow‐up period of 4.6±2.7 years. Blood‐based biomarkers (Aβ_42/40_, pTau217, GFAP, and NfL) were measured using the SIMOA platform. AD‐signature cortical thickness and hippocampal volume were determined using the longitudinal FreeSurfer pipeline. We used two linear mixed models to investigate the associations between baseline biomarker levels or biomarker slopes, and changes in AD‐signature cortical thickness and hippocampal volume over time.

**Result:**

SCD A+ showed greater decreases over time in AD‐signature cortical thickness (β_Time*Amyloid status_:‐0.04±0.01) and hippocampal volume (β:‐0.07±0.01) compared to SCD A−; both *p* <0.05). Higher baseline GFAP and increases in GFAP over time were associated with greater decreases in AD‐signature cortical thickness (β:‐0.02±0.01; β:‐0.33±0.10) and hippocampal volume over time (β:‐0.02±0.01; β:‐0.43±0.12; all *p* <0.01). Higher baseline pTau217 and increases in pTau217 were associated with greater decreases in hippocampal volume (β:‐0.03±0.01; β:‐0.97±0.44; *p* <0.05), but not in AD‐signature cortical thinning. Higher baseline NfL was associated with greater decreases in AD‐signature cortical thickness (β:‐0.01±0.01) and hippocampal volume (β:‐0.02 ± 0.01; *p* <0.05) over time, but changes in NfL were not. Neither baseline nor changes in Aβ_42/40_ were associated with atrophy.

**Conclusion:**

These findings suggest that gray matter atrophy in individuals with SCD is associated with AD‐related pathology as measured by blood‐based biomarkers, especially GFAP. This highlights the potential of blood‐based biomarkers as monitoring tools for structural brain changes and disease progression.